# Operating Point Self-Regulator for Giant Magneto-Impedance Magnetic Sensor

**DOI:** 10.3390/s17051103

**Published:** 2017-05-11

**Authors:** Han Zhou, Zhongming Pan, Dasha Zhang

**Affiliations:** College of Mechatronics Engineering and Automation, National University of Defense Technology, Changsha 410073, China; zhouhan10@nudt.edu.cn (H.Z.); zhangsha1024@163.com (D.Z.)

**Keywords:** GMI sensor, feedback control, compensation network, bias magnetic field

## Abstract

The giant magneto-impedance (GMI) magnetic sensor based on the amorphous wire has been believed to be tiny dimensions, high sensitivity, quick response, and small power consumption. This kind of sensor is usually working under a bias magnetic field that is called the sensor’s operating point. However, the changes in direction and intensity of the external magnetic field, or the changes in sensing direction and position of the sensor, will lead to fluctuations in operating point when the sensor is working without any magnetic shield. In this work, a GMI sensor based on the operating point self-regulator is designed to overcome the problem. The regulator is based on the compensated feedback control that can maintain the operating point of a GMI sensor in a uniform position. With the regulator, the GMI sensor exhibits a stable sensitivity regardless of the external magnetic field. In comparison with the former work, the developed operating point regulator can improve the accuracy and stability of the operating point and therefore decrease the noise and disturbances that are introduced into the GMI sensor by the previous self-regulation system.

## 1. Introduction

Micro magnetic sensors based on the amorphous wires have attracted much attention in the last decades [1,2]. This kind of magnetic sensor is based on the giant magneto-impedance (GMI) effect, which is a large change in the electrical impedance of soft magnetic materials upon the applied external magnetic field [3,4]. They have many advantages, like the tiny dimensions, high sensitivity, quick response, and low power consumption [5]. Since then, there are a lot of researches about the GMI sensor. Kuzminski et al. have improved the GMI effect with various thermal and mechanical treatments [6]. Das et al. have focused on the effect of different driving conditions on the rapidly quenched amorphous wires [7]. Aktham et al. have designed a GMI sensor based on the fully digital excitation, detection, and conditioning circuit [8]. T. Uchiyama et al. have designed a pico-Tesla resolution magneto-impedance sensor and applied it in biomagnetic field detection [9]. An operating point set by a bias magnetic field is required for the GMI sensor, which can make sure that the GMI sensor is working in a selected linear region with the highest sensitivity [10]. However, the changes in direction and intensity of the external magnetic field or the changes in sensing direction and position of the GMI sensor will influence the operating point. It may cause the fluctuation in sensitivity of the GMI sensor. Consequently, to maintain the sensor operation at a stable operating point is very important in the design of a highly sensitivity GMI sensor.

The traditional approaches are fixing the sensing direction of the GMI sensor perpendicular to the uniform geomagnetic field in the measurement or compensating the vector components of the uniform geomagnetic field with a permanent bias [11]. However, these two methods are unsuitable for the unstable external magnetic field or the moving GMI sensor. Then a feedback control based a GMI sensor was proposed [12]. This kind of GMI sensor uses a GMR sensor combined with the feedback control to construct a self-regulating system (SRS) to maintain the operating point of the GMI sensor on a uniform position. However, the performance of this GMI sensor is actually corresponding to the performance of GMR sensor and feedback control in SRS, because the operating point is used as a reference in the measurement of GMI sensor. Therefore, any improper design in SRS may introduce noise and disturbances into the GMI sensor. This shortcoming can decrease the sensor’s sensitivity. In order to overcome this problem and achieve a stable operating point for the GMI sensor, an operating point self-regulator based on the compensated feedback control is developed in this work. Experiment results show that the developed regulator can achieve a stable and accurate operating point. In comparison with the SRS-based GMI sensor, the proposed GMI sensor achieves a similar sensitivity and a much lower noise level at the same time.

The rest of paper is organized as the follows. Section 2 introduces the structure of GMI sensor, the mathematical model and the design of operating point self-regulator; Section 3 introduces the experiment procedures, results, and discussions. Section 4 describes the developed GMI sensor, and future work is also outlined.

## 2. The Structure of GMI Sensor

### 2.1. The Structure of Magnetic Detector

**Figure 1:**
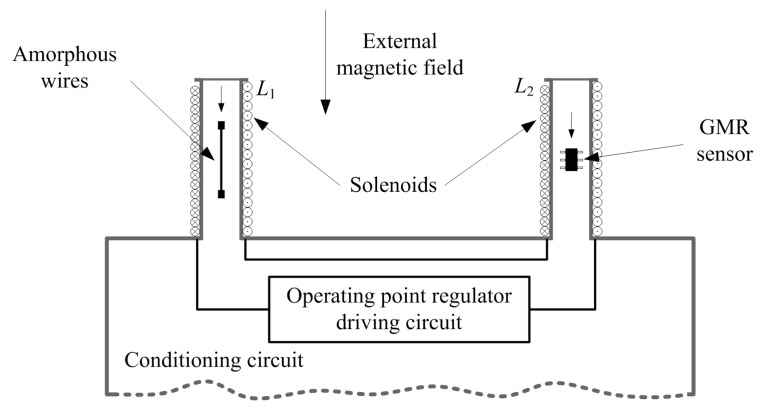
The structure of magnetic detector.

The structure of magnetic detector is shown in Figure 1. One glass-coated Co_71.8_Fe_4.9_Nb_0.8_Si_7.5_B_15_ amorphous wire is employed as the sensing elements with a length of 10 mm and a diameter of 30 μm. This wire is provided by the Advanced Technology & Materials Co. Ltd., Beijing, China. It is fixed on a printed circuit board with the silver conducting resin. Two solenoids (*L*_1_ and *L*_2_) are fabricated by an enameled Cu wire wrapped around the column of bobbins which are made by polytetrafluoroethylene (PTFE). The winding number and the length of two solenoids are both 1050 turns and 50 mm respectively. The solenoids are used to generate the bias magnetic field. The amorphous wire is placed in *L*_1_, while a GMR sensor is placed in *L*_2_. The GMR sensor is used to measure the DC or quasi-DC components in external magnetic field. The amorphous wire and the GMR sensor have the same sensing direction. *L*_1_ and *L*_2_ are connected in series and designed to generate a same magnetic field, like Figure 1 depicted. The purpose of this design is to protect the amorphous wire from the electronic magnetic fields produced by the signal and power wires of the GMR sensor. When the amorphous wire and the GMR sensor are put in the same solenoid, the outputs and the power supply wires of the GMR sensor must be placed very close to the amorphous wire. This can cause disturbances in the measurement results of amorphous wire. The GMR sensor is a commercial spin valve sensor with the type of SpHDE that be produced by the SpinIC Co. Ltd., Shanghai, China. It has a resolution of 2.7 nT and a classical sensitivity of 28.58 mV/V/mT. The supply voltage is +3.3 V and the supply current is about 6.0 mA.

The operating point self-regulator includes the GMR sensor, an amplifier, a second-order low-pass filter, a comparator, a V/I converter, and the solenoids. It will be described in Section 2.3.

### 2.2. The Design of GMI Sensor

**Figure 2:**
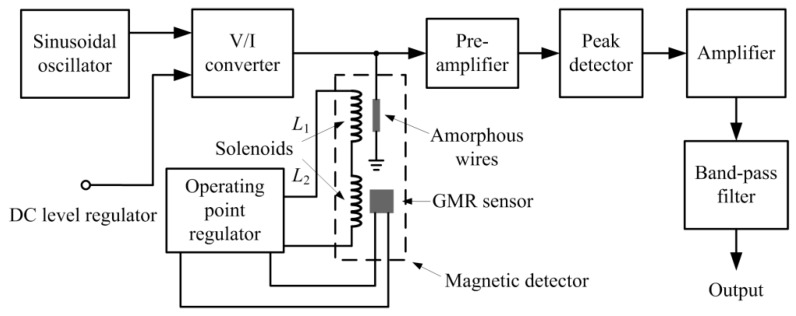
Block diagram of the developed GMI sensor.

Figure 2 shows the block diagram of the GMI sensor, which includes the sinusoidal oscillator, the dc level regulator, the voltage to current (V/I) converter, the magnetic detector as mentioned in Section 2.1, the pre-amplifier, the peak detector, the low-noise amplifier, the band-pass filter, and the operating point regulator which will be described in Section 2.3 [13,14]. When the amorphous wire is excited with a high frequency signal, a large change appears in the AC impedance of the amorphous wire because of the GMI effect, and this change subjects to an applied DC axial magnetic field [15]. The changes in AC impedance of the wire can be measured through the voltage across the amorphous wire. Here, we measure the changes in amplitude of the voltage signal to reflect the changes in its AC impedance.

**Figure 3:**
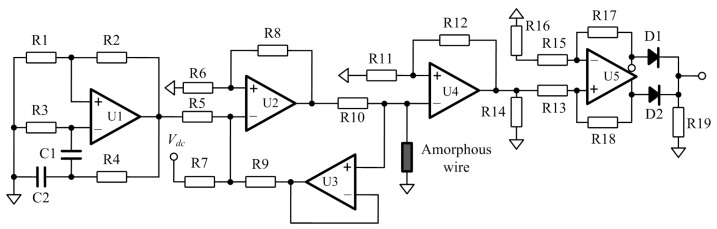
The GMI sensor circuit schematic.

The detailed circuits of GMI sensor is depicted in Figure 3. The excitation signal is produced with a sine-wave Wein-bridge oscillator and a V/I converter. A dc bias current is added into the excitation signal to expand the range of GMI sensor [16]. After the excitation signal passing through the amorphous wire, the voltage across the wire is firstly pre-amplified with the high speed feedback amplifier LM6172. Then a high speed full-wave rectifier based on the AD8132 is utilized to pick up the amplitude changes [17]. Afterwards, the weak amplitude signal is processed with the dual-channel filters SR650 produced by Standford Research Systems. Finally, this signal is connected to a data recording system for further processing.

If the magnetic field that is sensed by the GMR sensor and the amorphous wire are represented as ${\vec{B}}_{G}$ and ${\vec{B}}_{W}$ respectively, then they can be represented as

(1) $$\left\{ \begin{array}{l} {\vec{B}}_{W}={\vec{B}}_{e}+{\vec{B}}_{b}+{\vec{B}}_{e}^{\prime }+\Delta {\vec{B}}_{G}\\ {\vec{B}}_{G}={\vec{B}}_{b}+{\vec{B}}_{e}^{\prime } \end{array}\right.$$

where ${\vec{B}}_{e}^{\prime }$ is the DC or quasi-DC component in external magnetic field, ${\vec{B}}_{e}$ is the AC component in external magnetic field, ${\vec{B}}_{b}$ is the magnetic field that generated by the solenoids, and $\Delta {\vec{B}}_{G}$ is the noise magnetic field that produced by the GMR sensor and the regulation circuits. Here, ${\vec{B}}_{b}+{\vec{B}}_{e}^{\prime }$ is the bias magnetic field for the amorphous wire, which is maintained on a uniform position with the operating point regulator. In Equation (1), it is clear that the measurement results of amorphous wire are relying on the operating point that is decided by the operating point regulator and the GMR sensor.

It should be noticed that the sinusoidal current with a DC bias passing through the amorphous wire can also induce an AC current in the coil wound on the wire, which is the off-diagonal GMI effect [18,19]. However, the influence of this induction current is small because that its frequency is much higher than the bandwidth of the operating point regulator.

### 2.3. The Operating Point Self-Regulator and the Passive Phase-Lag Compensation Network

**Figure 4:**
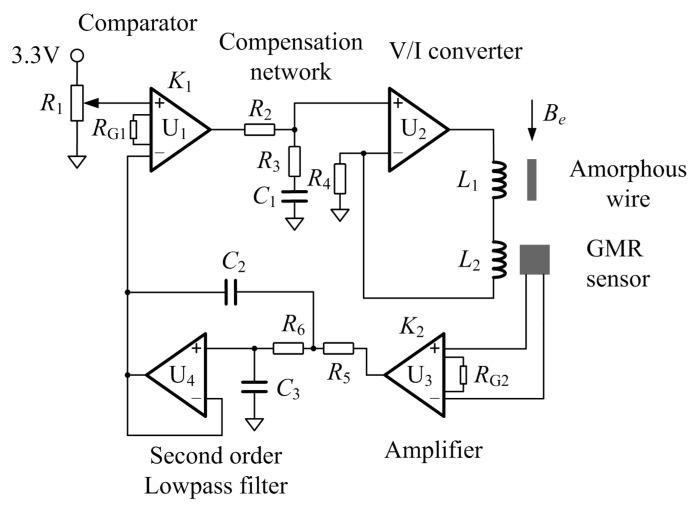
The structure of operating point self-regulator.

**Figure 5:**
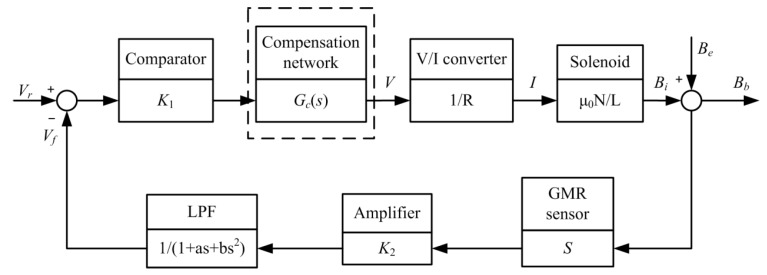
The block diagram model of the operating point feedback control.

The structure of proposed operating point self-regulator is presented in Figure 4. The operating point of GMI sensor is controlled with a reference voltage, a comparator based on the instrumentation amplifier INA128 and a V/I converter based on the operational amplifier AD8639. A feedback control based on the GMR sensor, the instrumentation amplifier INA128 and the second-order low-pass filter based on the operational amplifier AD8639 is applied to maintain the operating point on a uniform position. Its block diagram model is illustrated in the Figure 5 with an open loop transfer function like

(2) $$G(s)H(s)=\frac{K_{1}\mu_{0}N}{RL}\cdot \frac{K_{2}S}{1+as+bs^{2}}$$

where *μ*_0_ is the magnetic permeability of the vacuum, *N* and *L* are the winding number and length of the solenoids respectively, *K*_1_ and *K*_2_ are the gains of comparator and amplifier, *R* is the resistor that can regulate the current in V/I converter, *S* is the sensitivity of GMR sensor, and 1/(1 + *as* + *bs*^2^) is the transfer function of second-order low-pass filter.

**Figure 6:**
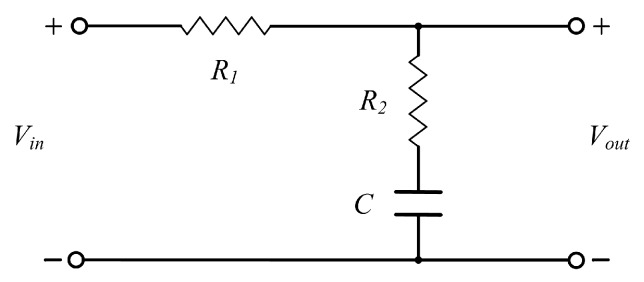
The phase-lag compensation network.

Generally, the operating point regulator based on the feedback system is controlled with the gain *K*_1_ [10]. In order to achieve an accurate operating point, the steady-state error of the feedback control system should be small. Thus the *K*_1_ needs to be large when the system is stable. Nevertheless, an increase in *K*_1_ results in an attendant decrease in the damping ratio of the system and therefore a more oscillatory response to a step input [20]. It means that the requirement of small steady-state error may cause fluctuations in the operating point when the external magnetic field is suddenly changing. In order to improve the performance of the operating point regulator and achieve an accurate and stable operating point, a phase-lag compensation network is added into the operating point regulator, like Figure 4 and Figure 5 show. The phase-lag network can provide an attenuation and increase the steady-state error constant of the feedback network [20]. There are two reasons for this compensation network in the design: firstly, the phase-lag network is utilized to increase the error constant and then reduce the steady-state error; secondly, the phase-lag network decreases the system bandwidth, which can suppress the high frequency noise. The phase-lag compensation network is depicted in Figure 6. Its transfer function *G_c_*(*s*) can be written as

(3) $$G_{c}(s)=\frac{R_{2}Cs+1}{\left(R_{1}+R_{2}\right)Cs+1}$$

when *τ* = *R*_2_*C* and *α* = (*R*_1_ + *R*_2_)/*R*_2_, then Equation (3) is

(4) $$G_{c}(s)=\frac{\tau s+1}{\alpha \tau s+1}=\frac{1}{\alpha }\frac{s+\frac{1}{\tau }}{s+\frac{1}{\alpha \tau }}$$

Generally, the phase-lag compensation network can be designed with the Bode diagram [20]. Consider the uncompensated system with the transfer function of Equation (2). *K*_2_ is 100 and *K*_1_ is adjustable; *R* is 392 Ω to provide a suitable current; *a* and *b* are 1.4142 and 1 respectively for a Butterworth second-order unity-gain low-pass filter. Therefore the uncompensated transfer function in Bode diagram is

(5) $$GH(j\omega )=\frac{K_{1}}{{\left(j\omega \right)}^{2}+1.414j\omega +1}$$

**Figure 7:**
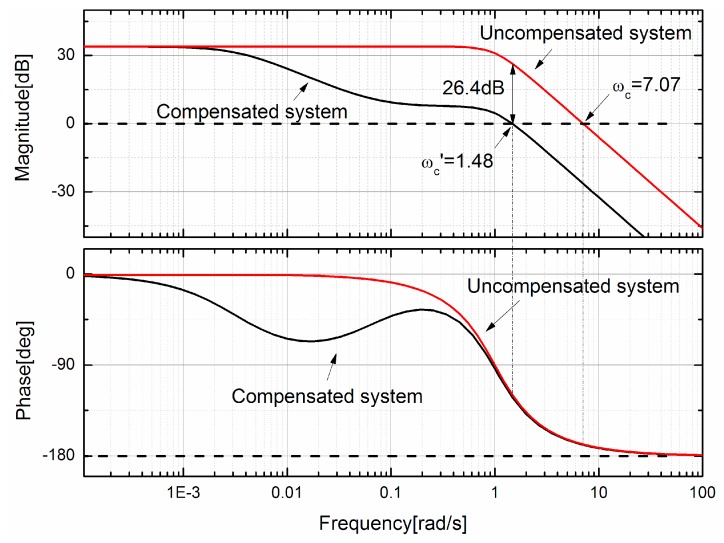
Design of the phase-lag network on the Bode diagram.

The target is to design a feedback system with the steady-state error less than 2%, while the percent overshoot for unit step input is less than 10%. It is easy to calculate that the steady-state error of the system when input is a unit step is

(6) $$e_{ss}=\frac{1}{1+K_{1}K_{2}\frac{\mu_{0}N}{RL}S}$$

with the known parameters, it is easy calculate that the *K*_1_ should be larger than 50. With this value, the Bode diagram of uncompensated system can be drawn as Figure 7 shows. It is obviously that the uncompensated system is a minimum phase system because all its zeros lie in the left-hand s-plane. Then with the Nyquist stability criterion, the uncompensated system is always stable because its phase is bigger than −180° [20], and its phase margin is *Φ*_pm_ = 11.5° at the crossover frequency *ω_c_* of 7.07. With its phase margin, the damping ratio *ζ* of the system is about 0.01 *Φ*_pm_ = 0.115. With the known damping ratio, the percent overshoot for a unit step input of the system is [20]

(7) $$P.O.=100e^{-\varsigma \pi /\sqrt{1-\varsigma^{2}}}$$

and the percent overshoot is about 69.5% with the gain control. In order to achieve an overshoot of less than 10%, the *ζ* should be about 0.6. Then the required phase margin of the compensated system is about *Φ*_pm_ = 60°, thus the new crossover frequency *ω_c_*’ is located where *Φ*(*ω*) = −120°. The new crossover frequency *ω_c_*’ is about 1.48 as the Bode diagram shows. The attenuation necessary to cause *ω_c_*’ to be the new crossover frequency is equal to 26.4 dB. Both the compensated and uncompensated magnitude curves are an asymptotic approximation. Because the attenuation is 26.4 dB, thus 20 log(1/*α*) = −26.4 dB, and the *α* is 20.9. Therefore the zero is 20.9 below the crossover, or 1/*τ* = *ω_c_*’/20.9 = 0.07, and the pole is at 1/*ατ* = 0.0033. The compensated system is then

(8) $$G_{c}(j\omega )GH(j\omega )=\frac{14.1j\omega +1}{299j\omega +1}\frac{50}{{\left(j\omega \right)}^{2}+1.414j\omega +1}$$

The frequency response of the compensated system is also shown in Figure 7. It is evident that the phase lag introduces an attenuation that lowers the crossover frequency and therefore increases the phase margin. As a final check, the phase margin at *ω_c_*’ = 1.46 is *Φ*_pm_ = 58.6°, which is the desired result.

## 3. Results and Discussion

### 3.1. The Perfromance of the Operating Point Self-Regulator

**Figure 8:**
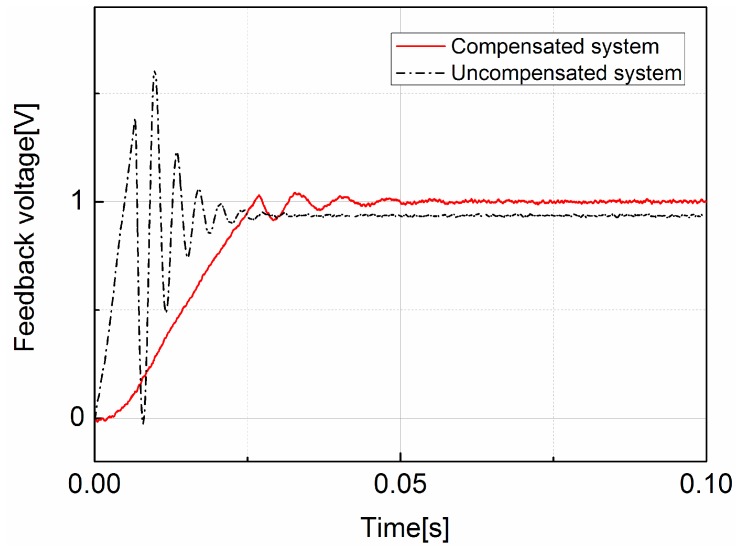
Time response to a step input for the uncompensated feedback control and the compensated feedback control.

The performance of the compensated operating point self-regulator was evaluated with a unit step input. Firstly, the regulator was set with a unit step input. Then the step responses of compensated and uncompensated regulator are recorded and compared. The results are depicted in Figure 8. As the results show, the compensated regulator has a much longer settling time in comparison with the uncompensated regulator, but its step overshoot and steady-state error are much smaller than the uncompensated regulator. The numerical results are presented in Table 1. This result can prove that the designed operating point self-regulator is stable, and the phase-lag network has improved the low-frequency performance of the regulator in comparison with the uncompensated regulator. This advantage can eliminate the fluctuations in the operating point when the external magnetic field is changing or the GMI sensor is placed on a moving platform. However, its cost is a longer settling time.

### 3.2. The Decision of the Operating Point

**Figure 9:**
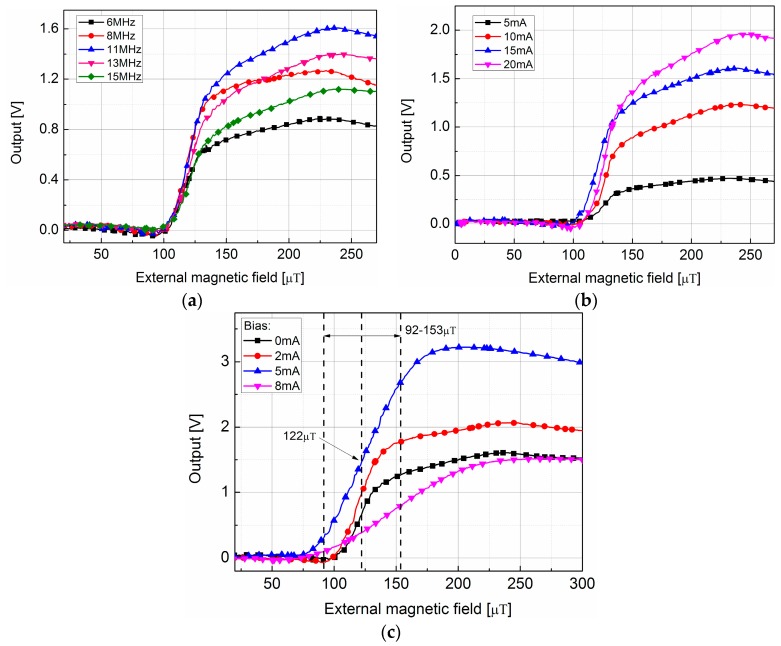
Sensor output voltages versus the external magnetic field characteristics. The results show comparisons between excitations with (**a**) different frequencies; (**b**) different amplitudes; (**c**) different DC bias.

For the purpose of comparison and convenient, the operating point regulator that designed in Section 2.3 will be called the compensated self-regulating system (CSRS). Firstly, the external magnetic field (${\vec{B}}_{e}$) versus the impedance characteristics of amorphous wire is measured to choose a suitable operating point. ${\vec{B}}_{e}$ was generated with a solenoid. The amorphous wire was placed in the center of solenoid, and its sensing orientation was parallel to the axis of solenoid. The dependences of the sensor output voltage on the ${\vec{B}}_{e}$ with different excitations are illustrated in Figure 9. All of these curves were measured without the CSRS and the amplification factor was about 30. The excitation signal was a sinusoidal signal with different frequencies, amplitudes, and DC bias [21]. The trend of the output voltage firstly increases to the maximum and then decreases as the external magnetic field gets larger. The increasing region is generally used as the operating region. As shown in Figure 9a, the 11 MHz excitation signal has the maximum slope, which means the highest sensitivity of amorphous wire. In Figure 9b, though the excitation signals with amplitude of 20 mA (peak-to-peak value) seems to have the maximum slope, but the 15 mA is chosen in the design for the purpose of decrease the power consumption. Figure 9c shows the AGMI effect in amorphous wire which is introduced by the DC bias in excitation signal. With the results in Figure 9c, the 5 mA DC bias is applied in the design to achieve the broadest linear region. Finally, based on the overall consideration of the sensitivity, consumption, and linearity, the excitation condition was chosen as: 11 MHz sinusoid with the amplitude of 15 mA, and the DC bias in excitation signal is 5 mA. The characteristic curve under this condition is drawn in Figure 9c (DC bias = 5 mA). In this curve, the linear region is about 92–153 μT. Then the operating point can be decided as the center of this region, which is about 122 μT. The operating point is marked in Figure 9c.

The operating point is controlled with the measurement of GMR sensor, and it is a spin valve sensor as mentioned in Section 2.1. However, this kind of sensor has a significant hysteretic characteristic, which can influence the regulation of the operating point. Therefore, the hysteretic characteristic of the GMR sensor was measured to evaluate its influence on the operating point of GMI sensor. This experiment was based on the GMR sensor with an external magnetic field generated by a Helmholtz coil. The range of external magnetic field was set from 43 μT to 248 μT to cover the linear range of the GMI sensor. The experiment results are presented in Table 2.

As the results in Table 2 show, the largest error in voltage that introduced by hysteretic is about 8.1 mV, which corresponds to the error of 0.86 μT in the operating point. Therefore the GMR hysteretic will cause an error of about 0.7% in the operating point of GMI sensor.

### 3.3. The GMI Sensor with the Compensated Self-Regulating System

**Figure 10:**
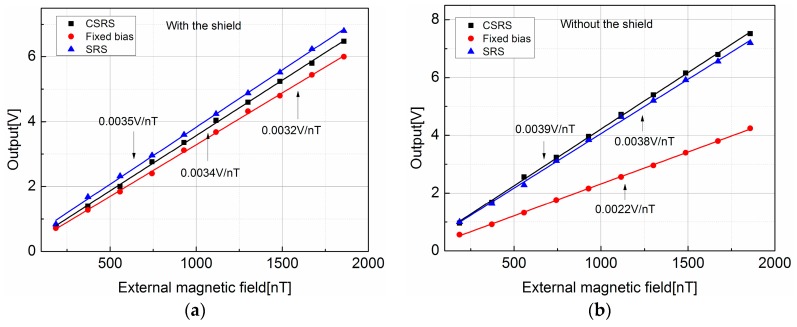
Output characteristic curves of the GMI sensor, (**a**) with the magnetic shield; (**b**) without the magnetic shield.

With the excitation conditions and the operating point that decided in Section 3.2, then the sensitivity of CSRS-based GMI sensor was measured and compared with that of the SRS-based GMI sensor and the fixed bias magnetic field GMI sensor. Firstly, their sensitivities were measured with a shield from the external magnetic field. The operating point was adjusted to 122 μT. The amplification factor in this experiment was about 60 dB while the cut-off frequency of low-pass filter was 15 Hz. The sensitivities were measured with a Helmholtz coil which was driven by an AC source. The frequency of the excitation current was 10 Hz, and its peak-to-peak value was adjusted to generate different sinusoidal magnetic field. The peak-to-peak values of the GMI sensor outputs were recorded, which are depicted in Figure 10a. The sensitivities were calculated based on the linear fit of these recorded data. As it shown, the sensitivities of CSRS-based sensor, SRS-based sensor, and fixed bias sensor are 0.0034 V/nT, 0.0035 V/nT, and 0.0032 V/nT respectively. It can be found that with the same operating point, the sensitivities of these kinds of GMI sensors are almost the same.

Then the sensitivities were measured without the shield. In this condition, the operating point is shift as the influence of external magnetic field. However, the SRS-based sensor and the CSRS-based sensor can regulate their operating point to adjust to the external magnetic field, while the fixed bias sensor cannot. With the measurement, the operating point of the SRS-based sensor and CSRS-based sensor are 116 μT and 120.4 μT respectively, and the operating point of fixed bias sensor is 80.2 μT. With the characteristic curve in Figure 9c, it is clearly that the sensitivity of fixed bias sensor will decrease. As the results depicted in Figure 10b, the sensitivities of the SRS-based and CSRS-based sensor are 0.0038 V/nT and 0.0039 V/nT respectively, which are similar to the results in Figure 10a. However, the sensitivity of the fixed bias sensor decreases to 0.0022 V/nT, just like in the analysis.

With this experiment, it can be proved that the CSRS and SRS can maintain the operating point and the sensitivity of GMI sensor in a uniform position regardless of the external magnetic field. However, the operating point of the fixed bias sensor changes with the external magnetic field, which causes the output of GMI sensor to be unstable.

**Figure 11:**
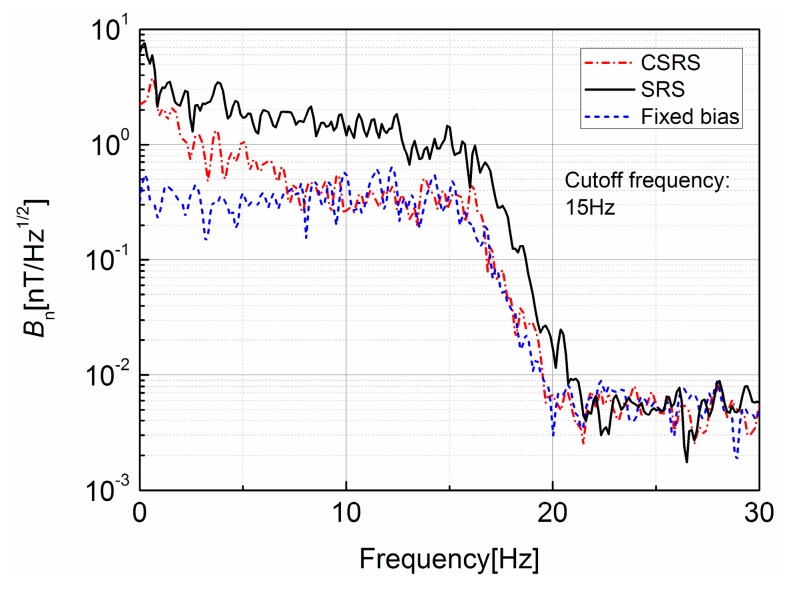
The noise spectral density of GMI sensors.

Then the noise of designed GMI sensor was measured. This measurement was carried out with the shield. The noise spectral density is illustrated in Figure 11. The amplification factor was about 60 dB while the cut-off frequency of low-pass filter was 15 Hz, and the sampling rate and resolution of the AD converter system were 10 ms and 16 bits, respectively. It is noted that the noise level of fixed bias sensor is about 0.38 nT/Hz^1/2^ in the pass band. The noise level of SRS-based sensor is much higher than the fixed bias sensor, which is about 2.9 nT/Hz^1/2^ from 0 to 5 Hz and about 1.6 nT/Hz^1/2^ from 5 to 12 Hz. This comparison can prove that the feedback control on the operating point introduces noise into the GMI sensor. The proposed CSRS-based sensor has a much higher noise level than the fixed bias sensor at the range from 0 to 7 Hz, which is about 2 nT/Hz^1/2^, but the phase-lag compensator has attenuated its noise to the same level of fixed bias sensor in 7 to 15 Hz as presented in Figure 11. With this experiment, it can be found that the compensation network decreases the system bandwidth and then suppresses the high frequency noise in the GMI sensor introduced by the feedback control.

Though the phase-lag network can decrease the noise level in SRS-based GMI sensor, it cannot suppress the noise in a low frequency region. This result can be explained, as in Figure 6, in that the phase-lag network has no effect in the low frequency region. In order to eliminate the low frequency noise in a SRS-based GMI sensor, a differential structure GMI sensor will be developed in the future work.

**Figure 12:**
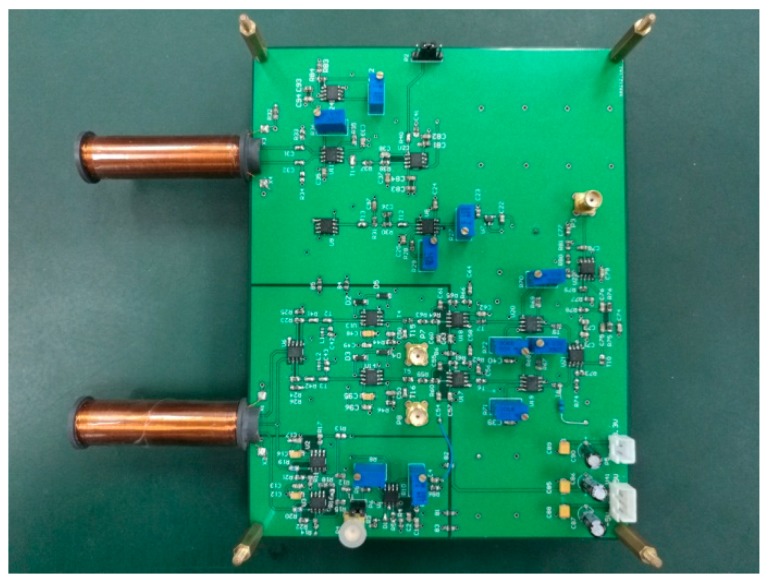
Photograph of the developed GMI sensor.

The entity of the GMI sensor is illustrated in Figure 12.

## 4. Conclusions

A GMI magnetic sensor based on the operating point self-regulator is developed in this work. The operating point self-regulator is based on a GMR sensor combined with a feedback control network. This regulator can maintain the operating point of GMI sensor on a uniform position regardless of the external magnetic field. In comparison with the former work, we have taken the noise and disturbances introduced into the GMI sensor by the operating point regulator into consideration. As the experiment shows, the developed operating point regulator has a steady-state error of about 2% and step overshoot of 4.2% at the same time. The optimum operating point of the GMI sensor was decided upon with several experiments, and the influence of hysteretic characteristic of the GMR senor on the operating point was discussed, too. With the optimum operating point and the operating point self-regulator, the developed GMI sensor exhibits a sensitivity of about 0.0034 V/nT in the AC magnetic field amplitude range from 0–2 μT. Comparisons with the SRS-based GMI sensor prove that the proposed GMI sensor achieves a similar sensitivity and a much lower noise level at the same time than the SRS-based GMI sensor.

Because the developed GMI sensor has a high sensitivity and a stable performance, it is suitable for the measurement of weak anomaly signals in low frequency changing external magnetic fields. Future work will concentrate on reducing the noise level of the developed sensor to achieve a high resolution.

## Figures and Tables

**Table 1:** The performance of two feedback control systems.

	Uncompensated System	Compensated System
Controller	Gain, *K*_1_	Phase-lag network
Step overshoot	72.2%	4.2%
Settling time (milliseconds)	23	35
Steady-state error for step	5.6%	2%

**Table 2:** The hysteretic characteristic of GMR sensor in an external magnetic field from 43 μT to 248 μT.

**Magnetic Field (μT)**	**43.4**	**62**	**80.6**	**99.2**	**117.9**	**136.5**
Output (increase) (V)	0.4346	0.5207	0.6086	0.699	0.7987	0.8890
Output (decrease) (V)	0.435	0.5260	0.6125	0.7026	0.7989	0.8909
**Magnetic Field (μT)**	**155**	**173.7**	**192**	**210.9**	**229.5**	**248.1**
Output (increase) (V)	0.9827	1.0834	1.1790	1.2768	1.3773	1.4887
Output (decrease) (V)	0.9843	1.0846	1.1833	1.2849	1.3834	1.4887
